# A genome-wide association and prediction study in grapevine deciphers the genetic architecture of multiple traits and identifies genes under many new QTLs

**DOI:** 10.1093/g3journal/jkac103

**Published:** 2022-04-29

**Authors:** Timothée Flutre, Loïc Le Cunff, Agota Fodor, Amandine Launay, Charles Romieu, Gilles Berger, Yves Bertrand, Nancy Terrier, Isabelle Beccavin, Virginie Bouckenooghe, Maryline Roques, Lucie Pinasseau, Arnaud Verbaere, Nicolas Sommerer, Véronique Cheynier, Roberto Bacilieri, Jean-Michel Boursiquot, Thierry Lacombe, Valérie Laucou, Patrice This, Jean-Pierre Péros, Agnès Doligez

**Affiliations:** AGAP Institut, Univ Montpellier, CIRAD, INRAE, Institut Agro, 34398 Montpellier, France; UMT Géno-Vigne, 34398 Montpellier, France; Université Paris-Saclay, INRAE, CNRS, AgroParisTech, GQE—Le Moulon, 91190 Gif-sur-Yvette, France; UMT Géno-Vigne, 34398 Montpellier, France; IFV, 30240 Le Grau-du-Roi, France; AGAP Institut, Univ Montpellier, CIRAD, INRAE, Institut Agro, 34398 Montpellier, France; UMT Géno-Vigne, 34398 Montpellier, France; AGAP Institut, Univ Montpellier, CIRAD, INRAE, Institut Agro, 34398 Montpellier, France; UMT Géno-Vigne, 34398 Montpellier, France; AGAP Institut, Univ Montpellier, CIRAD, INRAE, Institut Agro, 34398 Montpellier, France; UMT Géno-Vigne, 34398 Montpellier, France; AGAP Institut, Univ Montpellier, CIRAD, INRAE, Institut Agro, 34398 Montpellier, France; UMT Géno-Vigne, 34398 Montpellier, France; AGAP Institut, Univ Montpellier, CIRAD, INRAE, Institut Agro, 34398 Montpellier, France; UMT Géno-Vigne, 34398 Montpellier, France; AGAP Institut, Univ Montpellier, CIRAD, INRAE, Institut Agro, 34398 Montpellier, France; IFV, 30240 Le Grau-du-Roi, France; UMT Géno-Vigne, 34398 Montpellier, France; IFV, 30240 Le Grau-du-Roi, France; UMT Géno-Vigne, 34398 Montpellier, France; IFV, 30240 Le Grau-du-Roi, France; SPO, Univ Montpellier, INRAE, Institut Agro, 34060 Montpellier, France; SPO, Univ Montpellier, INRAE, Institut Agro, 34060 Montpellier, France; SPO, Univ Montpellier, INRAE, Institut Agro, 34060 Montpellier, France; SPO, Univ Montpellier, INRAE, Institut Agro, 34060 Montpellier, France; AGAP Institut, Univ Montpellier, CIRAD, INRAE, Institut Agro, 34398 Montpellier, France; UMT Géno-Vigne, 34398 Montpellier, France; AGAP Institut, Univ Montpellier, CIRAD, INRAE, Institut Agro, 34398 Montpellier, France; UMT Géno-Vigne, 34398 Montpellier, France; AGAP Institut, Univ Montpellier, CIRAD, INRAE, Institut Agro, 34398 Montpellier, France; UMT Géno-Vigne, 34398 Montpellier, France; AGAP Institut, Univ Montpellier, CIRAD, INRAE, Institut Agro, 34398 Montpellier, France; UMT Géno-Vigne, 34398 Montpellier, France; AGAP Institut, Univ Montpellier, CIRAD, INRAE, Institut Agro, 34398 Montpellier, France; UMT Géno-Vigne, 34398 Montpellier, France; AGAP Institut, Univ Montpellier, CIRAD, INRAE, Institut Agro, 34398 Montpellier, France; UMT Géno-Vigne, 34398 Montpellier, France; AGAP Institut, Univ Montpellier, CIRAD, INRAE, Institut Agro, 34398 Montpellier, France; UMT Géno-Vigne, 34398 Montpellier, France

**Keywords:** GWAS, genomic prediction, grapevine, *Vitis vinifera* L, genotyping-by-sequencing, yield components, secondary metabolites, genetic architecture, candidate genes

## Abstract

To cope with the challenges facing agriculture, speeding-up breeding programs is a worthy endeavor, especially for perennial species such as grapevine, but requires understanding the genetic architecture of target traits. To go beyond the mapping of quantitative trait loci in bi-parental crosses, we exploited a diversity panel of 279 *Vitis vinifera* L. cultivars planted in 5 blocks in the vineyard. This panel was phenotyped over several years for 127 traits including yield components, organic acids, aroma precursors, polyphenols, and a water stress indicator. The panel was genotyped for 63k single nucleotide polymorphisms by combining an 18K microarray and genotyping-by-sequencing. The experimental design allowed to reliably assess the genotypic values for most traits. Marker densification via genotyping-by-sequencing markedly increased the proportion of genetic variance explained by single nucleotide polymorphisms, and 2 multi-single nucleotide polymorphism models identified quantitative trait loci not found by a single nucleotide polymorphism-by-single nucleotide polymorphism model. Overall, 489 reliable quantitative trait loci were detected for 41% more response variables than by a single nucleotide polymorphism-by-single nucleotide polymorphism model with microarray-only single nucleotide polymorphisms, many new ones compared with the results from bi-parental crosses. A prediction accuracy higher than 0.42 was obtained for 50% of the response variables. Our overall approach as well as quantitative trait locus and prediction results provide insights into the genetic architecture of target traits. New candidate genes and the application into breeding are discussed.

## Introduction

With the 2 major challenges facing perennial fruit crops in general and grapevine in particular, i.e. decreasing phytosanitary products such as fungicide treatments and adapting to climate change, harnessing existing genetic diversity (Wolkovich *et al.* 2018) and breeding new varieties (Adam-Blondon *et al.* 2011) are both important levers. For the latter, many studies aimed at deciphering the genetic architecture of traits of interest by mapping quantitative trait loci (QTLs) in bi-parental progenies (Vezzulli *et al.* 2019). However, this approach suffers from several drawbacks: the limited allelic diversity in parents, the low number of recombination events in the progeny, the upward bias of estimated QTL effects, and the underestimation of the polygenic contribution for prediction purposes (Cardon and Bell 2001). As a result, all traits currently involved in grapevine marker-assisted selection (Vezzulli *et al.* 2019) are controlled by major genes, such as resistance to downy and powdery mildews (Di Gaspero *et al.* 2007), black rot (Rex *et al.* 2014), sex (Picq *et al.* 2014), berry color (Fournier-Level *et al.* 2009), seedlessness (Mejía *et al.* 2011), and Muscat aroma (Duchêne *et al.* 2009).

To overcome these limits, a few genome-wide association studies (GWASs) have been performed in cultivated grapevine diversity panels but, due to various reasons, failed to identify many new QTLs. Several articles (Myles *et al.* 2011; Zarouri 2016; Migicovsky *et al.* 2017; Laucou *et al.* 2018) harnessed phenotypic data from genetic resources repositories collected without a proper experimental design. Moreover, the first 3 articles cited used at most 10k SNPs despite the low extent of linkage disequilibrium (LD) (Myles *et al.* 2011; Nicolas *et al.* 2016). Among other articles, Zhang *et al.* (2017) focused on a single binary trait with a major QTL, seedlessness; Yang *et al.* (2017) used only 187 SSRs and 96 genotypes; Sargolzaei *et al.* (2020) focused on disease resistance using an 18K SNP microarray; Naegele *et al.* (2021) used at most 14k SNPs obtained by sequencing.

Moreover, most of these articles as well as one using 32k SNPs obtained with sequencing (Guo *et al.* 2019) used only SNP-by-SNP models. However, multi-SNP models have the advantage of explicitly assuming a genetic architecture, be it sparse with few major QTLs or dense with many small-effect QTLs, allowing them to benefit from a potential gain in power (Zhang *et al.* 2019). Furthermore, the effects of QTLs are often overestimated (Xu 2003) which leads to poor prediction (Meuwissen *et al.* 2001). Multi-SNP models provide a straightforward way to efficiently perform genomic prediction (de los Campos *et al.* 2013), notably for traits devoid of major QTLs.

Consequently, our objective was to perform whole-genome association and prediction for various traits of interest in grapevine breeding, likely to display different genetic architectures. We aimed at finding out to what extent genetic variation contributes to phenotypic variation, how it is organized in sparse and dense genetic components, how accurate genomic prediction might be, and which genes are present under the QTLs uncovered. Our approach builds on a large diversity panel of 279 *Vitisvinifera* L. cultivars (Nicolas *et al.* 2016) defined from the French collection of grapevine genetic resources and overgrafted in the vineyard in 5 randomized complete blocks. The panel was phenotyped over several years and under different conditions for 127 traits, including yield components, organic acids, aroma precursors, polyphenols, and a water stress indicator, which, along with 25 derived variables, totaled 152 response variables. The cultivars were genotyped with both microarray and sequencing after a reduction of genomic complexity (genotyping-by-sequencing, GBS), reaching a total of 63k SNPs. QTL detection and genomic prediction were then performed with multi-SNP models assuming different genetic architectures, and positional candidate genes were searched for under QTLs.

## Materials and methods

### Plant material and field trial

The panel of 279 cultivars of *Vitisvinifera* L. was designed to limit relatedness (any pair of cultivars in the panel corresponds to distinct genotypes with no parent in common) and is weakly structured in 3 genetic groups (Nicolas *et al.* 2016). In 2009, at the Domaine du Chapitre of Institut Agro Montpellier (Villeneuve-lès-Maguelone, France), the 279 cultivars as well as a control (cv. Marselan) were all overgrafted onto 6-year-old Marselan vines, itself grafted on rootstock Fercal, in a complete randomized block design with 5 blocks (A to E, Supplementary Fig. 1). Because of failed overgrafting, precocious death or fertility issues, only 270 cultivars out of the 279 in the whole panel could be phenotyped. The density of the field trial was 3,300 plants/ha (1 m between plants along the same rank and 2.5 m between ranks). Each of the 5 blocks contained 1 plant of each panel cultivar as well as a regular mesh of over-grafts of Marselan as control (between 23 and 39 per block). The double-cordon training system was applied.

A random subset of 21 full-sib genotypes of a Syrah × Grenache progeny, together with the 2 parents, was also used to assess out-of-sample genomic prediction. The field design had 2 random complete blocks established in 2003 as already described (Doligez *et al.* 2013). Each block contained the whole progeny (191 offsprings, one subplot each) as well as both parents (9 subplots each). Each subplot contained 5 grafted plants of the same genotype.

### Phenotyping

Here, we will use the term “trait” for any plant feature for which raw data were collected, whatever the year and condition. However, in our analyzes we use the term “response variable” because: (1) for some traits, data were acquired in different years and conditions, and hence analyzed separately; (2) we also combined several traits to define new variables. In the end, we thus analyzed 152 response variables from 127 traits.

In 2011 and 2012, the trial was not irrigated, and all the plants of the panel cultivars and controls were concurrently phenotyped. For each plant, we recorded the number of clusters (NCBLU) and harvested 3 clusters at 20°Brix, which provided the sampling date (SAMPLDAY, in days since January 1). We recorded mean cluster weight (MCW, in g), mean cluster length (in cm), mean cluster width (in cm), and cluster compactness (from 1 to 9 on the OIV 204 scale; OIV, 2009). One hundred berries randomly sampled from the central third of clusters were weighed, providing the mean berry weight (MBW, in g). In the winters of 2011–2012 and 2012–2013, the number of woody shoots (NBWS) and pruning weight (PRUW, in kg) were recorded for each plant. In 2011, the veraison date (onset of ripening, VER, in days since January 1) was also recorded. Two variables were computed from these traits: the veraison-maturity interval (VERMATU as SAMPLDAY—VER, in days), and plant vigor (VIG as PRUW/NBWS, in kg). In 2011 and 2012, juices were made from the sampled berries and analyzed to measure δ^13^C (D13C) as previously detailed (Pinasseau, Vallverdú-Queralt, *et al.* 2017). In 2012 were also determined glucose (GLU), fructose (FRU), malate (MAL), tartrate (TAR), shikimate (SHI), and citrate (CIT) concentrations, all in μEq l^−^^1^, as previously described (Rienth *et al.* 2016). Six variables were computed from these traits: the sum of glucose and fructose (GLUFRU), glucose divided by fructose (GLUONFRU), malate divided by either tartrate (MALTAR), shikimate (SHIKTAR), or citrate (CITAR) and the sum of glucose and fructose (GLUFRUTAR).

In 2014 and 2015, irrigation was applied to blocks C, D, and E only (Pinasseau, Vallverdú-Queralt, *et al.* 2017), and only panel cultivars were phenotyped. As above, 3 clusters per plant were harvested at 20°Brix, providing the MCW (in g). Berries sampled from different blocks with the same water treatment were pooled per cultivar. More details on berry sampling and processing, as well as polyphenols and δ^13^C measurements and analysis are described elsewhere (Pinasseau, Vallverdú-Queralt, *et al.* 2017). From the data available on the 105 different polyphenols in µg per berry (Pinasseau, Verbaere, *et al.* 2017), a few typos were corrected and 17 extra variables were calculated (Pinasseau, Vallverdú-Queralt, *et al.* 2017). In addition, 2 aroma precursors, β-damascenone (BDAM, in μg l^−^^1^) (Kotseridis *et al.* 1999) and potential dimethyl sulfide (PDMS, in μg l^−1^) (Segurel *et al.* 2005) were also quantified. The volume and weight of juice samples were recorded, allowing to assess their effects when included as cofactors in the statistical analyses.

A total of 127 traits were phenotyped, from which 25 extra variables were computed. Because irrigation was applied to some blocks only in 2014–2015, the few traits phenotyped both in 2011–2012 and in 2014–2015 were analyzed separately. Overall, 152 response variables were analyzed (Supplementary Tables 1 and 2).

The sanitary status of cultivars regarding the presence of 5 viruses (CNa, GLRaV1, GLRaV2, GLRaV3, and GFkV) was assessed by ELISA from plants at INRAE Vassal (Marseillan, France). Flower sex (OIV 151) and berry skin color (OIV 225) of each panel cultivar were retrieved from (Laucou *et al.* 2018) and completed with the database of INRAE Vassal germplasm repository (https://bioweb.supagro.inra.fr/collections_vigne/Home.php?l=EN).

Berry weight was phenotyped on the Syrah × Grenache cross in 2005, 2006, and 2007 in the same way as on the panel (Doligez *et al.* 2013), except that 8 clusters per genotype and per block were harvested instead of 3.

### Genotyping

#### Data acquisition and analysis of microarray SNPs

The panel and Syrah × Grenache progeny were genotyped with the GrapeReSeq 18k Vitis Illumina microarray (Laucou *et al.* 2018). Data processing (see Supplementary Text 1, Supplementary Figs. 2, 3, and Supplementary Table 2) resulted in 13,925 SNPs for 277 cultivars. After filtering on LD above 0.9 and minor allele frequency (MAF) below 0.05, 10,530 SNPs remained (see Supplementary Fig. 4), thereafter referred to as the “microarray-only” SNPs.

#### Data acquisition and analysis of sequencing SNPs

The panel was also genotyped by sequencing (GBS, Elshire *et al.* 2011). Keygene NV owns patents and patent applications protecting its Sequence Based Genotyping technologies. Data processing consisted in read checking with FastQC version 0.1.2 (Andrews 2016), demultiplexing with a custom script, cleaning, and trimming with CutAdapt version 1.8.1 (Martin 2011), alignment on the PN40024 12Xv2 reference sequence (Canaguier *et al.* 2017) with BWA MEM version 0.7.12-r1039 (Li 2013) and realignment with GATK version 3.7 (DePristo *et al.* 2011), followed by variant and SNP calling with GATK HaplotypeCaller, and a final filtering step, notably to discard SNP genotypes with <10 reads or quality below 20 (see Supplementary Text 2 and Supplementary Table 3). It resulted in 184,145 SNPs with <30% missing data for the 279 panel cultivars.

#### Joint imputation of microarray and GBS SNPs

The 13,925 microarray SNPs and 184,145 GBS SNPs for 277 common cultivars were combined into a set of 197,885 common SNPs (after duplicate removal) using coordinates on the 12Xv2 reference sequence (Canaguier *et al.* 2017). Missing data were imputed using LD with Beagle version 4.1-r862 (Browning and Browning 2009) as advised by Swarts *et al.* (2014)^,^ with window = 1,000, overlap = 450, ne = 10,000, and otherwise default parameters. After filtering for LD above 0.9, 90,007 SNPs remained (see Supplementary Fig. 3), and while subsequent filtering for MAF below 0.05 resulted in 63,105 SNPs (see Supplementary Fig. 4), thereafter referred to as the “microarray-GBS” SNPs. We also imputed the Syrah × Grenache SNP genotypes similarly using Beagle.

### Statistical modeling of phenotypic data

We performed a 2-stage analysis of each response variable using univariate regression models. In the first stage, estimates of total genotypic values were obtained (detailed in this section). In the second stage (see next section), these were regressed on SNP genotypes to identify QTLs, estimate their allelic effects and assess prediction accuracy.

To decrease the influence of potential outliers, all polyphenols (the compounds as well as the calculated variables) had their raw data automatically transformed with the natural log. For the other traits, when their raw phenotypic data were too skewed as visually assessed, they were also log-transformed (see Supplementary Fig. 5 and Supplementary Table 4).

#### Assessment of spatial heterogeneity

In 2011–2012, phenotypic data for the control were spatially analyzed (Cressie 1993) in a way similar to Hamann *et al.* (2002). First, a global linear model was fitted with R/stats with fixed effects for block, year, block–year interaction, PRUW, NBWS, vigor, and all 5 viruses (PRUW and vigor were discarded when vigor itself was the response). Facilitated by R/MuMIn version 1.40 (Bartoń 2017), model comparison was performed by maximum likelihood (ML), the best model being selected based on the corrected Akaike information criterion, AICc (Burnham and Anderson 2004). For each year separately, the empirical variogram of residuals from the best model was computed, on which several variogram models were fitted by ML with R/gstat version 1.1.5 (Pebesma 2004): exponential, spherical, gaussian, and Stein’s parametrization of the Matérn model. The variogram model with the smallest sum of squared errors was then used to perform spatial interpolation by kriging, i.e. best linear unbiased prediction (BLUP) of the control’s response variable at all locations. By visually assessing the slope of the best variogram model fitted to the empirical variogram (Supplementary Fig. 6) and the prediction errors from cross-validation (data not shown), it was concluded that there was no need to correct for spatial heterogeneity.

In 2014–2015, the control was not phenotyped, an irrigation treatment was applied, and samples from different blocks with the same irrigation level were pooled (Pinasseau, Vallverdú-Queralt, *et al.* 2017), hence preventing the assessment of any potential spatial heterogeneity as above.

#### Estimation of genotypic values

For each response variable, a global linear mixed model was defined with multiple fixed effects [for the 2011–2012 data set: block, year, block–year interaction, PRUW, NBWS, vigor, and all 5 viruses, PRUW and vigor being discarded when vigor itself was the response; for the 2014–2015 data set: irrigation, year, irrigation–year interaction, °Brix (as there can be small deviations from 20°Brix)], and all 5 viruses, as well as the volume and weight of juice samples for BDAM and PDMS) together with 2 random effects (genotype and genotype–year interaction). The global model was fitted by ML with R/lme4 version 1.1.19 (Bates *et al.* 2015). The output was given to R/lmerTest version 3.1-2 (Kuznetsova *et al.* 2017) to use its function “step.” Backward elimination of random-effect terms was performed using likelihood ratio test, followed by backward elimination of fixed-effect terms using *F*-test for all marginal terms, i.e. terms that can be dropped from the model while respecting the hierarchy of terms in the model, with a 0.05 *P*-value threshold for both types of terms. The final model after backward elimination was then refitted by restricted ML (ReML) to obtain unbiased estimates of variance components and empirical BLUPs of genotypic values. The acceptability of underlying assumptions (homoscedasticity, normality, independence) was visually assessed by plotting residuals and BLUPs. Broad-sense heritability on a genotype–mean basis (H^2^) was computed using 2 estimators. The first assumes a balanced design (Falconer and Mackay 2009): $H_{C}^{2}$ = σ^2^_g_/[σ^2^_g_ + (σ^2^_g:__y_/*n*_y_) + (σ^2^_e_/(*n*_y_ × *n*_r_)] where σ^2^_g_ is the variance of the genotypic values, σ^2^_g:__y_ is the variance of the genotype–year interactions, *n*_y_ is the arithmetic mean number of trials (years), σ^2^_e_ is the variance of the errors (residuals), and *n*_r_ the arithmetic mean number of replicates per trial. The second estimator, $H_{O}^{2}$, allows for unbalanced data (see Oakey *et al.* 2006, for details). Robust confidence intervals for variance components, heritability and genotypic coefficient of variation were obtained by parametric bootstrap as recommended by Schweiger *et al.* (2016), using the percentile method (Carpenter and Bithell 2000) in the R/lme4 and R/boot packages. In the Syrah × Grenache progeny, empirical BLUPs of genotypic values for berry weight were obtained in the same way.

### Statistical modeling of genotypic data

#### Genetic architecture assumed sparse

We used 2 types of models to perform genome-wide association testing and detect QTLs. The first is the SNP-by-SNP model as implemented in GEMMA version 0.97 (Zhou and Stephens 2012). For each SNP p, eBLUP(**g**) = **1 **μ + M_a,__p_ β_p_ + **u **+** e** where eBLUP(**g**) is a vector of responses of length *N*, M_a,__p_ is a vector of length *N* with the genotypes at the pth SNP (additive coding), β_p_ is its effect modeled as fixed, **u** ∼ *N*_N_(**0**, σ_u_^2^ A) is a vector of length *N* corresponding to a polygenic effect modeled as random where the covariance matrix A contains additive genetic relationships (Vitezica *et al.* 2013), and **e** ∼ *N*_N_(**0**, σ_e_^2^ Id) with *N* the Normal distribution of dimension *N*, **0** a vector of zeros, and Id the identity matrix of dimension N×N. eBLUPs of **g** were used instead of BLUEs as they are known to be more accurate for prediction and selection purposes, notably thanks to the shrinkage property (Piepho *et al.* 2008). Our goal was to test the null hypothesis β_p_=0 while controlling for relatedness between genotypes. Controlling the family-wise error rate at 5% to account for multiple testing, the effect of an SNP was deemed significant when the *P* value from the Wald test statistic was lower than the Bonferroni threshold.

The second type of models jointly analyzes all SNPs with the goal of selecting a subset of those with large effects while handling LD. This SNP selection can be achieved in a frequentist setting via stepwise regression (Segura *et al.* 2012). It starts with the SNP-by-SNP model, followed by inclusion, at every iteration, of the SNP with the smallest *P* value as an additional fixed effect, until the proportion of variance explained by the polygenic effect is close to zero. The SNP effects deemed significant were those of the best model selected according to the extended BIC. We fitted it with R/mlmm.gwas v1.0.4 (Bonnafous *et al.* 2018) allowing a maximum of 50 iterations. SNP selection can also be achieved in a Bayesian setting with the following model: eBLUP(**g**) = **1 **μ + M_a_**β** + **e**, where M_a_ is a NxP matrix of SNP genotypes (additive coding), with the so-called spike-and-slab prior for each SNP p, β_p_ ∼ π_0_ δ_0_ + (1 - π_0_) N_1_(0, σ_β_^2^), δ_0_ being a point mass at zero. We fitted it with the variational algorithm, faster than MCMC, implemented in R/varbvs version 2.5.7 (Carbonetto and Stephens 2012). An SNP was deemed significant when its posterior inclusion probability, PIP_p_ = Pr(β_p_ ≠ 0), was higher than 0.80.

Beyond this focus on statistical significance (McShane and Gal 2017), we provide all estimates of significant additive SNP effects with a quantification of their uncertainty (Supplementary Table 5).

#### QTL definition and annotation

QTLs were defined as intervals around significant SNPs based on LD decay (Bonnafous *et al.* 2018) (see Supplementary Text 3). A comparison was made between the QTLs detected in this study and (1) a first list of already-published QTLs (Vezzulli *et al.* 2019), significant at a 5% genome-wide threshold, that were classified according to the Vitis INRAE ontology v2 (https://urgi.versailles.inra.fr/ephesis/ephesis) and slightly edited for automatic processing (see Supplementary Text 3); and (2) a second list of significant hits from a few GWAS publications after converting their coordinates on the genome reference we used.

In terms of annotation, as a given locus can be a QTL for multiple response variables, we first merged our 489 reliable QTLs (found with at least 2 methods, see *Results*) across all response variables, which resulted in 134 distinct genomic intervals (Supplementary Table 9). These intervals had a median length of 100,001 kb (with a minimum of 100,001 kb and a maximum of 1,072,169 kb). We then searched for overlaps between them and the Vcost version 3 annotations totalizing 42,413 gene models from Canaguier *et al.* (2017), also using the correspondence between IGGP (International Grapevine Genome Program) and NCBI RefSeq gene model identifiers provided by the URGI (https://urgi.versailles.inra.fr/Species/Vitis/Annotations).

##### Genetic architecture assumed dense

To estimate the proportion of variance of empirical BLUPs of genotypic values explained by the cumulative contribution of SNPs (Yang *et al.* 2010) (PVE_SNPs_), we used the well-known multi-SNP model termed ridge regression (also known as “RRBLUP”) which assumes a dense architecture: eBLUP(**g**) = **1 **μ + M_a_**β** + **e** where **β** ∼ N_P_(**0**, σ_β_^2^ Id). It is known to be equivalent to the “GBLUP” model (Habier *et al.* 2007; Vitezica *et al.* 2013): eBLUP(**g**) = **1 **μ + **g_a_** + **e** where **g_a_** ∼ N_N_(**0**, σ_a_^2^ A) with A, the N×N matrix of additive genetic relationships, proportional to the matrix product M_a_$M_{a}^{T}$ once M_a_ is centered using allele frequencies. It is similar for the dominance genotypic values **g_d_** ∼ N_N_(**0**, σ_d_^2^ D) where D is the N×N matrix of dominance genetic relationships. Because the estimators of additive and dominance relationships from SNPs assume linkage equilibrium, a 0.5 LD threshold was applied when computing A and D. We fitted the models with R/lme4 and computed confidence intervals for variance components by bootstrap as above.

##### Genomic prediction

Out-of-sample prediction was assessed within the panel by 5-fold cross-validation repeated 10 times with R/caret version 6 (Kuhn 2018), using R/varbvs that assumes a sparse genetic architecture and R/rrBLUP version 4.5 (Endelman 2011) that assumes a dense architecture (infinitesimal model). Note that the QTL results from the GWAS analysis were not used when training each model, to avoid overfitting. We assessed prediction accuracy between empirical BLUPs of genotypic values and their predictions with various metrics: root mean square error, Pearson’s linear correlation coefficient (corP), Spearman’s rank correlation coefficient (corS), as well as outputs from the simple linear regression of observations on predictions such as the intercept, slope, adjusted coefficient of determination (*R*^2^), and the *P*-value of the test for no bias.

Out-of-sample prediction was also assessed by training rrBLUP and varbvs methods on the whole panel and predicting empirical BLUPs of genotypic values for the 23 genotypes of the Syrah × Grenache cross.

## Results

### Estimation of broad-sense heritability and genetic coefficient of variation

We took advantage of the *V.vinifera* L. panel of 279 cultivars suitable for GWAS and representing the INRAE Vassal germplasm repository to set up a randomized-complete-block field trial (Supplementary Fig. 1). It was phenotyped for 127 traits from which 25 extra variables were computed. All 152 response variables displayed substantial variation (Supplementary Fig. 5). For some polyphenol variables, part of the variation was obviously associated with skin color, 137 cultivars out of 279 having colored skin berries. When phenotyped, the control cultivar allowed us to establish that (1) part of this variation was due to genetic differences between panel cultivars (Supplementary Fig. 5), and that (2) spatial heterogeneity was negligible (Supplementary Fig. 6). The amount of missing data among response variables ranged from 15.78% to 43.93% (Supplementary Table 4). To account for such unbalance, we fitted linear mixed models and obtained BLUPs of genotypic values. After model selection, the final set of fixed and random effects differed between response variables (Supplementary Table 4), with year and genotype–year interaction effects being selected in most cases.

**Fig. 1.**
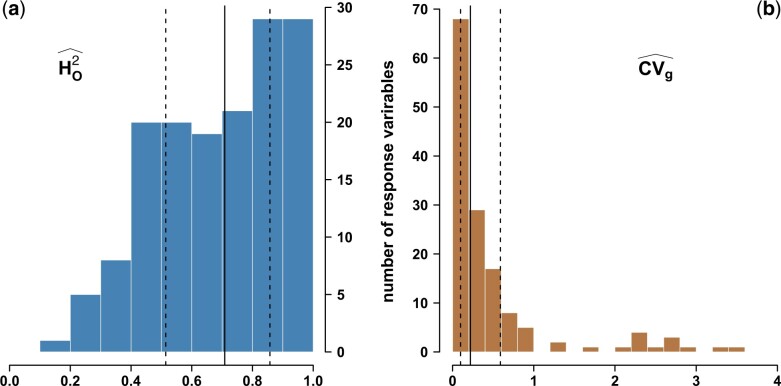
Estimation in a diverse panel of *Vitis vinifera* L. of (a) broad-sense heritabilities for 152 response variables using the estimator from Oakey *et al.* (2006), $H_{O}^{2}$, and (b) their genetic coefficients of variation, CV_g_. Vertical lines indicate the median (plain), and quantiles at 0.25 and 0.75 (dotted).

We then assessed the accuracy with which genotypic values were estimated using broad-sense heritability (the higher, the better). As shown in Fig. 1, 76.6% of broad-sense heritability estimates were above 0.5, with narrow confidence intervals (Supplementary Table 4). Two estimators, $H_{C}^{2}$ and $H_{O}^{2}$, handling missing data differently, gave very similar estimates (Supplementary Table 4), thus indicating that genotypic values of all cultivars were accurately estimated for most response variables. Moreover, 92.7% of the genetic coefficients of variation were above 5% and 59.1% above 20% (Fig. 1; Supplementary Table 4).

### Combining genotyping technologies to explain more genetic variance

We then aimed to explain the variance of these genotypic BLUPs with SNP genotypes. For that purpose, we used 2 sets of SNPs, the “microarray-only SNPs” (10,503 SNPs) and “microarray-GBS SNPs” (63,105 SNPs).

Because LD is known to be short in *V.vinifera* L. (Myles *et al.* 2011; Nicolas *et al.* 2016), we increased the SNP density initially obtained with the microarray by sequencing with complexity reduction (GBS). Raw reads had high quality along their sequences, although many displayed adapters’ content at their 5’ end, which had to be trimmed off. After demultiplexing, more than 95% of the reads were assigned to a cultivar. After alignment on the reference genome, the median coverage depth of regions having at least 1 read, averaged over cultivars, was 21.7, which allowed to accurately call both homozygous and heterozygous SNP genotypes after filtering out SNPs supported by <10 reads.

**Fig. 2.**
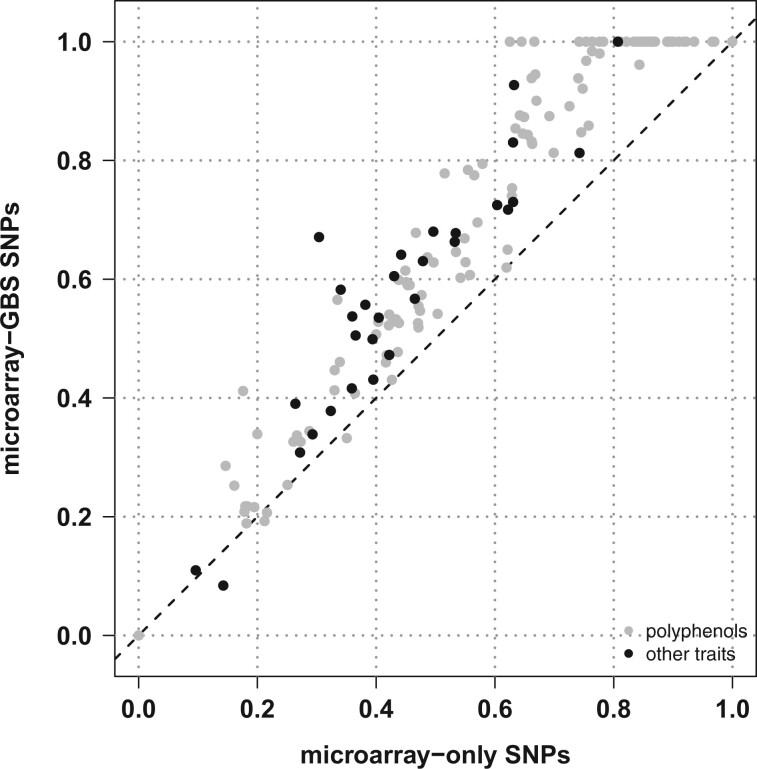
Estimation in a diverse panel of *Vitis vinifera* L. of the proportion of variance in genotypic BLUPs explained by SNPs for 152 response variables and 2 SNP densities, assuming an additive-only, polygenic architecture.

Compared with the microarray-only SNP set, the combined microarray-GBS set displayed a substantially higher SNP density along all chromosomes (Supplementary Fig. 4). We then estimated the additive genetic relationships between cultivars (Supplementary Fig. 7), confirming the weak structure in 3 subgroups corresponding to wine west, wine east, and table east. The matrix of genetic relationships was used to estimate the proportion of variance in genotypic BLUPs explained by SNPs (PVE_SNPs_). Assuming an additive-only, polygenic architecture, PVE_SNPs_ was higher with microarray-GBS SNPs than with microarray-only SNPs for 97.8% of responses variables (Fig. 2; Supplementary Table 5). This showed the advantage of combining SNPs so that more QTLs are in LD with at least 1 genotyped SNP.

Models with both additive and dominance relationships either failed to converge or then, only with difficulty, most probably because the matrix of dominance relationships was very similar to the identity matrix, making it indistinguishable from the error term (Supplementary Fig. 7).

### QTL detection by GWAS and identification of candidate genes

The GWAS methods used in the following were first checked on 2 previously phenotyped traits, flower sex and berry skin color, for which the already known genetic architecture consists in a major QTL. Results were coherent with the literature (Fournier-Level *et al.* 2009; Picq *et al.* 2014): a major QTL on chromosome 2 for flower sex (around coordinate 4,769,151) and for berry skin color (around coordinate 15,753,009). Other weaker QTLs were also found, on the Unknown chromosome for flower sex [note that Tello *et al.* (2019) found chunks of chromosome 2 in the Unknown chromosome when building genetic maps], and on chromosome 7 and 13 for berry skin color (consistent with QTLs for skin anthocyanidin content found by Guo *et al.* 2015).

Each response variable phenotyped in this study was analyzed with an SNP-by-SNP model to identify significant SNPs (Supplementary Table 6). For each response variable, QTLs were defined as LD-based intervals around each significant SNP (Supplementary Table 7), and then merged when overlapping (Supplementary Table 8). As summarized in Table 1, at least 1 QTL was found for 66.4% of response variables with the microarray-GBS SNPs, when compared with 57.9% with the microarray-only SNPs.

**Table 1.** Comparison between methods in terms of the number of QTLs (#QTLs) found in a diverse panel of *Vitis vinifera* L. for 2 SNP data sets, summed up over all response variables.

Method		Microarray- only SNPs			Microarray- GBS SNPs		
Model	Software	#RVs	#sSNPs	#QTLs	#RVs	#sSNPs	#QTLs
SNP-by-SNP	GEMMA	88	2,295	1,179	101	7,855	1,784
Multi-SNP	mlmm.gwas	148	1,257	1,243	125	703	692
	Varbvs	118	266	257	119	258	257

Also indicated are the number of response variables with at least one QTL (#RVs), and the number of significant SNPs (#sSNPs).

To benefit from a potential gain in power, we fitted 2 multi-SNP models that both provided more response variables with at least 1 QTL compared with the SNP-by-SNP model, whatever the SNP set (Table 1). Within multi-SNP methods, mlmm.gwas found more significant SNPs and QTLs than varbvs, and for more response variables. Yet, the interpretation is not straightforward as these methods do not use the same criterion for declaring an SNP as significant. Surprisingly, for mlmm.gwas, the numbers of response variables with at least 1 QTL, significant SNPs and QTLs were lower with more tested SNPs.

By summing over the 150 response variables with at least 1 QTL, a total of 3,490 QTLs were found (Supplementary Table 8), which corresponded to an increase of 196% in the number of QTLs and of 70% in the number of response variables with at least 1 QTL, compared with applying the SNP-by-SNP method on the microarray-only SNPs. Among these QTLs, 136 were found by all 3 methods, while 3,001 were found by a single method only and 1,598 by multi-SNP methods only (Supplementary Fig. 9). Furthermore, over these 150 response variables, 26 had no QTL according to the SNP-by-SNP method but at least one found by both multi-SNP methods (Supplementary Fig. 10).

All chromosomes harbored at least 1 QTL (Supplementary Fig. 11), and most QTLs found only by the multi-SNP mlmm.gwas method fell far from QTLs found by other methods (Supplementary Fig. 12). Moreover, 90% of the QTLs found only by the SNP-by-SNP method GEMMA clustered on chromosome 2 for 64 response variables, all of them being polyphenols, in relation with the anthocyanin-related MYB genes on this chromosome (Matus *et al.* 2008). This was expected because GEMMA ignores LD between SNPs. In contrast, the multi-SNP varbvs method was more parsimonious, yet had enough power to identify significant SNPs in regions in which GEMMA did not identify any signal (Supplementary Fig. 12).

**Fig. 3.**
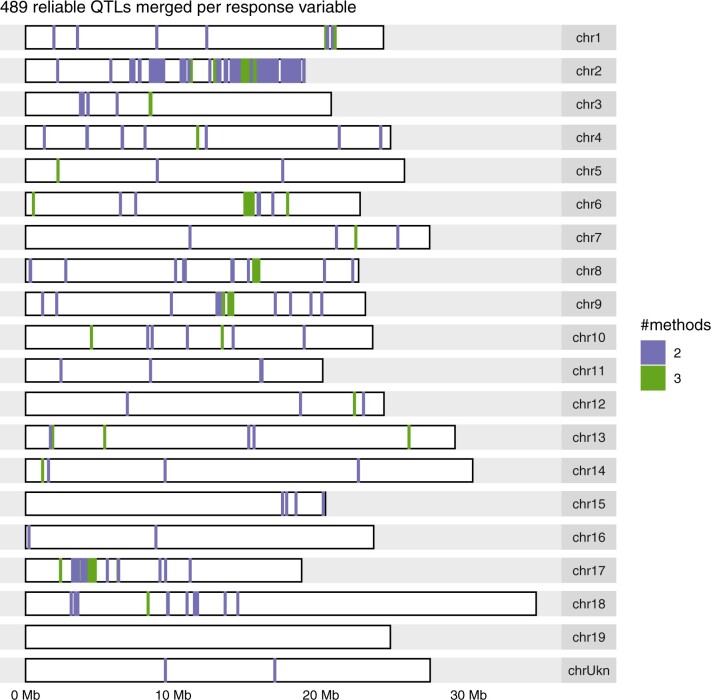
Genomic distribution of the most reliable QTLs identified by 2 methods in a diversity panel of *Vitis vinifera* L. after merging them over microarray-only and microarray+GBS SNP sets per response variable. The color legend indicates the number of methods that identified a given QTL.

To prioritize QTLs for further investigation, 489 QTLs involving 124 response variables were deemed reliable as they were found by at least 2 methods (Supplementary Table 9 and Supplementary Fig. 13). They corresponded to 59% less QTLs but 41% more response variables with at least 1 QTL, compared with applying the SNP-by-SNP method on the microarray-only SNPs. All chromosomes harbored at least 1 such reliable QTL, except chromosome 19 (Fig. 3).

The 489 reliable QTLs were compared with the largest list of QTLs detected in grapevine bi-parental crosses compiled so far (Vezzulli *et al.* 2019). Among the 22 traits in common, QTLs were found on the same chromosome for 7 only (Supplementary Table 8): cluster number (on chromosome 7), berry weight (on chromosomes 1, 2, 8, 11, 15, and 17), malate (on chromosomes 9 and 18), glucose to fructose ratio (on chromosome 2), mean degree of polymerization of tannins (chromosome 17), total concentration of native anthocyanins (on chromosome 2), and %B-ring methylated anthocyanins (on chromosome 2).

We also compared our reliable QTLs with significant GWAS hits published in grapevine. Only 2 traits (cluster and berry weight) were phenotyped in at least 1 other study with at least 1 significant GWAS hit found (Zarouri 2016; Laucou *et al.* 2018; Guo *et al.* 2019). For berry weight, out of the 10 QTLs we found, 8 were deemed new on chromosomes 1, 2, 8, 11, 15, and 17. We also found 2 QTLs on chromosome 8 close to a known GWAS hit (Zarouri 2016), but did not recover other hits on chromosomes 5, 17, 18, and 19 as in Zarouri (2016) and Guo *et al.* (2019). For cluster weight, we found 2 new QTLs on chromosomes 1 and 3 but did not recover other hits on chromosomes 5 and 13 (Zarouri 2016; Laucou *et al.* 2018).

The comparison of our reliable QTLs with the reference gene annotations detected 1,926 distinct gene models (Supplementary Table 10). Out of these, only 980 had a proposed putative function (Supplementary Table 11).

### Assessment of genomic prediction and insight into genetic architectures

**Fig. 4.**
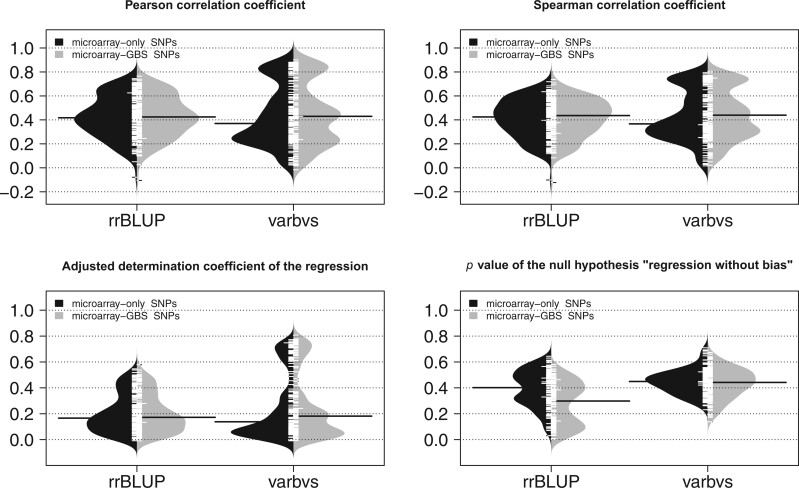
Assessment of genomic prediction accuracy within a diversity panel of *Vitis vinifera* L. by repeated 5-fold cross-validations, comparing 2 SNP sets (microarray-only and microarray-GBS) and 2 methods (rrBLUP assuming a dense genetic architecture and varbvs assuming a sparse genetic architecture) for 152 responses variables. The 4 displayed metrics were averaged over folds and replicates.

We assessed the accuracy of genomic prediction by cross-validation within the panel of 279 cultivars (Supplementary Table 12). We compared 2 methods assuming contrasted genetic architectures: additive infinitesimal for rrBLUP and additive sparse for varbvs. Both the median Pearson and Spearman correlation coefficients between observed and predicted genotypic values fell between 0.37 and 0.44, similarly for both SNP sets and methods (Fig. 4). These correlations showed substantial correlation with broad-sense heritability (Supplementary Fig.14), higher for varbvs (∼0.65) than for rrBLUP (∼0.54). However, the distributions of varbvs’ correlation coefficients were clearly multi-modal, with the majority being lower than rrBLUP’s but still a substantial fraction being higher. This confirmed the robustness of rrBLUP’s predictions irrespective of the underlying architecture (Wang *et al.* 2015). Yet varbvs can provide substantially better predictions than rrBLUP for traits for which the genetic architecture is likely to be rather sparse.

Moreover, rrBLUP results did not seem to depend on the SNP set whereas they were slightly better with the microarray-GBS SNPs for varbvs. This suggests that, among the extra SNPs provided by GBS, varbvs managed to identify those that improved its prediction accuracy. Concerning the *P* value of the test for no bias, varbvs showed similar values across both SNP sets, higher than rrBLUP in general and above 0.05, suggesting an absence of bias. On the contrary, rrBLUP showed lower *P* values with the microarray-GBS SNPs, suggesting that its assumption of all SNP effects being nonzero may be too strong for these traits, especially when SNP density is high.

We also used the 279 cultivars panel as a training set to predict MBW in a subset of a Syrah × Grenache progeny. With rrBLUP (respectively, varbvs), this gave a Pearson correlation of 0.56 (0.35), an adjusted coefficient of regression of 0.28 (0.08), and a *P* value of 1.6x10^−4^ (3.5x10^−3^) when testing for no bias. The correlation is particularly promising for rrBLUP compared with varbvs, in agreement with the results obtained by cross-validation within the panel (Pearson correlation of 0.71 with rrBLUP and 0.61 with varbvs).

Finally, combining results from both genome-wide association and genomic prediction studies provides insight into the genetic architecture of the studied traits. In Table 2, trait classes are sorted according to the following metric: the difference between the accuracy of genomic prediction assuming a sparse additive genetic architecture (as implemented in varbvs) vs a dense one (as implemented in rrBLUP), using the Spearman correlation coefficient from the cross-validation above as a proxy of prediction accuracy (Supplementary Table 13).

**Table 2:** Types of additive genetic architecture per trait class in a diversity panel of *Vitis vinifera* L. based on the accuracy of genomic prediction assuming a sparse genetic architecture (method “varbvs) or a dense one (method “rrBLUP”) over all response variables (RVs).

Trait class	#RVs	Median of cor_S_(varbvs)—cor_S_(rrBLUP)	Additive genetic architecture	#relQTLs	$H_{O}^{2}$
Biochemical	136	+0.05 [−0.12, +0.18]	Sparse (−)	3.0 [0.0, 8.0]	0.69 [0.41, 0.96]
Abiotic stress	2	−0.04 [−0.09, +0.02]	Dense (−)	0.5 [0.1, 0.9]	0.37 [0.21, 0.52]
Phenological	3	−0.04 [−0.06, −0.03]	Dense (+)	2.0 [0.4, 2.0]	0.80 [0.72, 0.83]
Morphological	5	−0.08 [−0.08, −0.07]	Dense (+)	1.0 [0.0, 1.6]	0.82 [0.74, 0.87]
Agronomical	6	−0.12 [−0.09, +0.19]	Dense (−)	1.5 [0.5, 5.0]	0.79 [0.38, 0.95]

Also indicated are a symbol for the confidence level in the classification (+ for high, − for low), the number of reliable QTL (#relQTLs) and the broad-sense heritability estimated according to Oakey et al. (2006) ($H_{O}^{2}$); for both, the median, quantile at 10% and quantile at 90 are given.

Overall, the median of this metric is positive only for response variables corresponding to biochemical traits (mostly polyphenols), suggesting they display a sparse genetic architecture. All other trait classes have a negative median metric, suggesting a dense genetic architecture. When taking into account the distribution of the metric, the classification in sparse or dense architecture is deemed more trustworthy when the quantile interval does not include 0, which is the case for phenological and morphological traits.

Apart from the abiotic stress variable δ^13^C, all response variables had a high median broad-sense heritability (around 0.7 and above), indicating a higher measurement quality, hence also contributing to increased trustworthiness in the suggested genetic architecture. Moreover, in the case of the biochemical response variables, the median number of reliable QTLs is higher than for the other trait classes, although there is a large variation. This is consistent with their genetic architecture deemed sparse, for which one expects to have QTLs with an effect large-enough to be found significant.

## Discussion

### Design and analysis of field trials for perennials

Acquiring phenotypic data from which genotypic values can be deduced with sufficient accuracy is a major challenge, especially because a large panel is a prerequisite to provide enough statistical power to detect QTLs (Nicolas *et al.* 2016). Our randomized block design certainly helped in reaching medium to high broad-sense heritability for most traits. Those with low heritability may be linked to the difficulty of sampling fruits at a similar physiological stage, a particularly pressing issue for grapevine due to the strong intra- and inter-cluster heterogeneity between berries (Shahood 2017). Automatizing new protocols (Bigard *et al.* 2018) remains to be done to phenotype large panels.

At the first stage of the analysis, we chose to include PRUW, the number of wooding shoots and vigor as explanatory factors in the global model, but neither flower sex nor berry color. Our rationale was that the former 3 are more influenced by the way the field trial is conducted than the latter 2, which are under a stronger genetic determinism (Fournier-Level *et al.* 2009; Picq *et al.* 2014). This approach would hence keep most genetically based variation between genotypes for the second stage of the analysis (genome-wide association and genomic prediction). More generally, this raises the question of how to deal with multiple traits to exploit their correlations (Supplementary Table 14 and Supplementary Fig. 15). Most multivariate linear models place all the traits on the same level, which complicates the understanding of their genetic architecture (Kemper *et al.* 2018). A more ambitious approach would leverage functional–structural plant models (Sievanen *et al.* 2014) but it notably requires the phenotyping of key phenological stages for the whole panel, as well as the nondestructive phenotyping of major physiological processes over time.

### Increase of genotyping density

Validating heterozygous SNP genotypes from GBS data is notoriously difficult (Swarts *et al.* 2014). We hence looked at the proportion of variance in BLUPs of genotypic values explained by SNP genotypes (PVE_SNPs_). The improvement obtained with the microarray-GBS set increased our trust in the genotyping and imputation procedures. Yet, PVE_SNPs_ did not equal 1 for all response variables. Several factors can underlie this discrepancy. First, empirical BLUPs of genotypic values are not fully accurate versions of the “true” genotypic values, as reflected by broad-sense heritability. Second, the microarray-GBS SNPs may not be in strong-enough LD with all “true” QTLs. The number of SNPs required might reach half a million in grapevine (Nicolas *et al.* 2016), a value likely to be similar in other perennial fruit crops with low LD. Moreover, many pan-genome structural variations could remain undetected, which calls for whole-genome sequencing (Marroni *et al.* 2014).

### Sensitivity and specificity of QTL detection

Our study which detected many reliable QTLs benefited from a highly favorable context combining a representative panel, an adequate experimental design and a large number of phenotyped traits. When comparing GWAS methods, a major misleading factor is LD, which SNP-by-SNP methods do not take it into account whereas multi-SNP methods do, albeit differently depending on the details of each method. We hence compared the 3 methods in terms of QTLs, defined here as intervals around significant SNPs, instead of significant SNPs directly. We used the genome-wide distribution of LD to define the extent of QTLs, which ignores local variations along the genome. Haplotype-based methods could provide complementary information, but is beyond the scope of this work.

We compared our reliable QTLs with those from the literature on bi-parental crosses passing a 5% genome-wide significance threshold. Therefore, when we deemed one of our QTLs new, it may have been found at a chromosome-wide significance threshold; nevertheless, it is reported as reliable for the first time in our study. This comparison could be achieved for a very small subset of common traits only. Part of the reason why may be that the traits studied here include an exhaustive list of polyphenols that have rarely been quantified elsewhere. In addition, we faced the notorious difficulty to assess whether the same trait acronym used in different articles indeed corresponded to the same biological trait. A wider usage of a trait ontology, such as the *Vitis* ontology, seems the only way forward (Krajewski *et al.* 2015).

Furthermore, when comparing our results on cluster and berry weights with those from the literature obtained by GWAS, we found discrepancies: several of our QTLs were new, and several QTLs reported by others were not found in our analysis. This may be due to 4 types of differences, (1) the composition of the association panels, (2) the genotyping densities, (3) the phenotyping protocols, and (4) the statistical models. Reanalyzing these data sets was out of the scope of this work but could be done in the future depending on data availability.

### Focus on some candidate genes

For various traits, our association study identified many QTLs (Supplementary Tables 8 and 9) containing numerous genes (Supplementary Tables 10 and 11). As such, this large database is of interest per se for further investigations. We chose to focus here our discussion on a subset of traits, i.e. phenolic compounds, organic acids and δ^13^C.

#### Candidate genes for phenolic compounds

Our results confirm known features of the genetic regulation of phenolic compounds in grape, such as the region on chromosome 2 containing the MybA genes cluster. It governs not only the amount and quality of anthocyanins, but also the traits concerning flavonols as already observed (Malacarne *et al.* 2015). Our study also confirms a large QTL for tannins composition, located on chromosome 17 and already detected in a Syrah × Grenache progeny (Huang *et al.* 2012), which contains the candidate gene *VvLAR2* (leucoanthocyanidin reductase, Vitvi17g00371). *LAR* was initially characterized as being able to catalyze the formation of catechin terminal units (Bogs *et al.* 2005), but it was demonstrated more recently that *VvLAR* could have an additional role in controlling the degree of polymerization (Yu *et al.* 2019).

Our study also identified new regions for already-studied traits, such as one involved in anthocyanin acylation and tri-hydroxylation on chromosome 13. This QTL was not detected neither in a Syrah × Pinot Noir progeny (Costantini *et al.* 2015), nor in a Red Globe × Muscat of Hamburg progeny (Sun *et al.* 2020), and is distinct from either the functionally validated anthocyanin acyltransferase on chromosome 3 (Rinaldo *et al.* 2015) and the Flavonoid 3ʹ,5ʹ-hydroxylase cluster located on chromosome 6. This region contains 2 *WRKY* transcription factors (Vitvi13g00189 and Vitvi13g01916) orthologuous of *AtWRKY55* and *AtWRKY54/70* (Wang *et al.* 2014*)*. *WRKY* transcription factor mediates stress responses in plants (Phukan *et al.* 2016), and *AtWRKY70* was also described to control JA‐induced anthocyanins accumulation (Li *et al.* 2006). In grape, anthocyanin acylation and hydroxylation are affected under abiotic stress (Ollé *et al.* 2011), thus these *WRKY* transcription factors appear as candidate genes to modulate anthocyanin composition.

Moreover, this is the first GWAS in grape for some phenolic compounds such as phenolic acids or dihydroflavonols. A region on chromosome 6 controlling the amount of astilbin, resulting from the rhamonsylation of taxifolin, contains 4 uncharacterized flavonoid O-glycosyltransferases (Vitvi06g01093, Vitvi06g01097, Vitvi06g01099, Vitvi06g01100) that could be involved in this reaction.

#### Candidate genes for organic acids and δ^13^C

No QTL for citrate had yet been found, but our study yielded one on chromosome 3. This 56-kb region contains several candidates’ genes: 5 copies of allene oxide synthase (Vitvi03g00391 to 5), and the long chain acyl coA synthase 2 (Vitvi03g00388). Oxylipins formed by allene oxide synthases are precursors of jasmonates (Farmer and Goossens 2019) involved in rewiring central metabolism, thus decreasing the levels of those metabolites associated with active growth such as citrate (Savchenko *et al.* 2019). Moreover, the closest homologue of the last gene in *Arabidopsis* participates in oil synthesis in seed endoplasmic reticulum, where its overexpression triggers the activation of genes involved in glycolysis (Ding *et al.* 2020). Acyl coA synthase 2 and citrate synthase may hence compete for AcetylCoA, which yields citrate when condensed with oxaloacetate.

Regarding malate, Vitvi09g00195 located on chromosome 9, possibly in a QTL found by Bayo-Canha *et al.* (2019) in a parental genetic map from a bi-parental progeny, encodes a chloroplastic glyoxylate/succinic semialdehyde reductase 2 which has 2 connections with malate synthesis. First, this enzyme may scavenge glyoxylate in the chloroplast matrix and protect photosynthesis from its adverse effects (Simpson *et al.* 2008). Glyoxylate is, with acetyl CoA, the direct substrate of malate synthase in glyoxysome and such diversion from the classical photorespiratory pathway was documented in *Chlorella* (Xie *et al.* 2016). Second, succinic semialdehyde dehydrogenase is the last enzyme in the gamma-aminobutyric acid shunt of the TCA cycle (Zarei *et al.* 2017), forming succinate that is readily oxidized to fumarate, the precursor of malate in mitochondria. In another new malate QTL on chromosome 12, Vitvi12g00505 encodes a cytosolic aconitate hydratase that may complement the activities of the mitochondrial and glyoxysomal ones, respectively, involved in the metabolism of dicarboxylate and glyoxylate. On chromosome 18, Vitvi18g01038 encodes V-type proton ATPase subunit a2, a part of the hydrophobic V0 rotor that generates the membrane potential essential for the storage of organic acids in grapevine fruit (Terrier *et al.* 2001). Noticeably, in a Riesling × Gewurztraminer progeny, subunits G of V-ATPase on chromosomes 8 and 13 were suggested as candidate genes for acidity QTLs (Duchêne *et al.* 2020).

Relevant candidate genes were also found under novel QTLs for δ^13^C, in particular Vitvi08g02203 on chromosome 8 that encodes the transcriptional regulator *TAC1*-like. In rice it corresponds to a major QTL controlling tiller angle, with a direct influence on leaf exposition to light (Yu *et al.* 2007). We also noticed the presence of different candidate genes involved in stele expansion or differentiation, such as *CASP-*like proteins and Lonesome Highway (*LHW*) transcription factor.

### Genomic prediction, and the wider goal of understanding genetic architectures

The accuracy of genomic prediction, assessed for the first time for such a large number of traits by cross-validation within a grapevine diversity panel, reached promising levels according to the median Pearson correlation (around 0.4), even though the coefficient of determination remains substantially lower (around 0.17, Fig. 4). Nevertheless, breeders mostly aim at accurately predicting the ranks of candidate genotypes, and the median Spearman correlation around 0.4 is relevant for that purpose.

Cross-validation results are interesting per se as they provide an upper threshold for prediction accuracy. Yet, the ultimate goal for breeders lies in training a model on a reference panel to predict genotypic BLUPs in a segregating population. When testing this with a subset of a Syrah × Grenache progeny not belonging to the panel, the accuracy metrics were lower than with the within-panel cross-validation, although they displayed the same trend in terms of methods. Ridge regression model (rrBLUP) performed better than the sparse regression model (varbvs), which may be due to the essentially infinitesimal architecture of the trait despite a few larger QTL segregating for this trait in the progeny (Doligez *et al.* 2013). This promising result was studied in more details with other traits and other progenies in grapevine (Brault *et al.* 2022), as well as in other perennial fruit crops (Minamikawa *et al.* 2017; Roth *et al.* 2020).

In terms of genetic architectures, we focused on additive ones and attempted to distinguishing trait classes with a sparse vs dense architecture. Leaving aside the trait class “abiotic stress” that had low broad-sense heritability, our results based on prediction accuracy indicated a sparser architecture for biochemical traits, vs a denser one for phenological, morphological and agronomical traits. In the framework of genotype–phenotype maps, this may correspond to the fact that biochemical traits are closer, in a causal sense, to genetic variation (such traits are sometimes called “endophenotypes”), hence making QTL detection easier. On the opposite, the other trait classes are more integrated, in the sense of resulting from multiple developmental and ecophysiological processes (Granier and Vile 2014). Moreover, the determination of genetic architecture is also known to depend on sample size and LD extent (Wimmer *et al.* 2013). In contrast to what is expected on annual plant breeding populations, we identified traits with better prediction accuracy assuming a sparse architecture rather than a dense one, in spite of the rather small sample size of our panel. This was likely due to the short LD within this diversity panel, a notable feature of perennial plants, although these results may not stand for grapevine bi-parental breeding populations with longer LD.

### Conclusion

This work demonstrated the feasibility of performing a GWAS in a perennial fruit crop such as grapevine for numerous, mostly complex traits related to various aspects of plant biology and breeding. A key ingredient for field trials remains the experimental design necessary to achieve high broad-sense heritability. We also provided dense genotyping data for further studies on the panel, although, given the low LD, an even higher number of SNPs would be advantageous. In terms of GWAS, we confirmed that a gain in power is possible when using multiple-SNP models. Overall, we identified new QTLs as well as promising genes under them, leading to mechanistic hypotheses yet to be tested. In terms of genomic prediction, we provided a distribution of prediction accuracy across many traits likely to have various genetic architectures. We confirmed the usage of the RRBLUP/GBLUP model assuming a dense architecture as a relevant default. Yet, we showed that a model assuming a sparse architecture can reach higher prediction accuracy for some traits, notably in the case of traits closer to the genetic variation. As such, our work provided important results for the contribution of genomic prediction to perennial crop species breeding.

## Ethical standards

The authors declare that the experiments comply with the current laws of the country in which they were carried out.

## Data availability

The data that support the findings of this study are openly available, for sequences at the NCBI as BioProject PRJNA489354 and, for all the rest, at the Data INRAE repository at https://doi.org/10.15454/8DHKGL. The code that supports the findings of this study is openly available at the Data INRAE repository at https://doi.org/10.15454/8DHKGL. Many of the custom functions we used are available in a R package for reproducibility purposes, rutilstimflutre (Flutre 2019).

Supplemental material is available at https://doi.org/10.15454/8DHKGL.
